# 3D‐MOF‐Lattice Inspired Programmable Metamaterials Based on Reconfigurable Polyhedral Origami

**DOI:** 10.1002/advs.202517921

**Published:** 2025-12-14

**Authors:** Xi Kang, Yangqin Zhang, Hongshuang Fan, Ziyan Xu, Yue Dong, Bing Li

**Affiliations:** ^1^ Guangdong Provincial Key Laboratory of Intelligent Morphing Mechanisms and Adaptive Robots Harbin Institute of Technology Shenzhen 518055 China; ^2^ School of Robotics and Advanced Manufacture Harbin Institute of Technology Shenzhen 518055 China

**Keywords:** mechanical metamaterials, metal‐organic frameworks (MOFs), polyhedral origami pattern

## Abstract

Metamaterials have attracted significant attention due to their unconventional mechanical properties. However, a major limitation of conventional metamaterials lies in the fixed and uniform microstructures, limiting their ability to realize diverse functionalities. Inspired by the network chemistry of the 3D architectures of metal–organic frameworks (MOFs) crystal networks, a reconfigurable design strategy based on polyhedral origami patterns is proposed. Leveraging intrinsic bifurcation behavior, a set of modular units with tunable stiffness and adjustable Poisson's ratios is developed. Both experimental and theoretical investigations confirm that these modules exhibit diverse mechanical responses, including quasi‐zero, positive stiffness, bistability, and a continuously tunable Poisson's ratio spanning negative to positive values. By assembling modules in desirable modes, a programmable metamaterial network with customizable mechanical performance is presented. This approach provides a versatile platform for designing multifunctional mechanical metamaterials and offers practical value in applications such as advanced shock‐absorbing systems, enhancing versatility and adaptability.

## Graphical abstract

A novel metamaterial design strategy: inspired by MOFs crystal networks, creating reconfigurable modular polyhedral units to overcome the limitations of traditional materials. These modules exhibit adjustable stiffness, bistability, and Poisson's ratio that can be adjusted from negative to positive values. This programmable network allows for customized mechanical performance, opening up new possibilities for advanced applications such as adaptive damping systems.

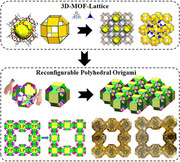

## 1 Introduction

Metal‐organic frameworks (MOFs) are constructed by connecting metal‐containing units with organic linkers using strong bonds (network synthesis).^[^ ^1^ ^]^ Their open crystalline frameworks with permanent and excellent porosity make MOF materials have a wide range of potential uses in gas storage, separation, energy storage, and catalysis. MOFs have attracted much attention due to their beautiful and diverse structures and wide applications, so the identification and description of the underlying topological network extracted from the experimental crystal structure of MOFs has emerged.^[^ ^2^ ^]^ The regularity of MOFs crystal networks in natural tiling can be generalized to combinations and arrays of polyhedral units. This generalization forms a fundamental basis for the independent design of metamaterial units.^[^ ^3^ ^]^ Metamaterials, artificially engineered to exhibit properties unattainable in natural materials, have significantly advanced materials science and engineering. Their extraordinary physical behaviors, which arise from precisely architected microstructures rather than inherent material composition, result in extraordinary characteristics such as special optical properties,^[^ ^4^, ^5^ ^]^ cloaking effects,^[^ ^6^, ^7^ ^]^ and tunable deformation ability.^[^ ^8^, ^9^, ^10^ ^]^ Over the past two decades, the field of metamaterials has experienced rapid expansion, with its applications extending to electromagnetics,^[^ ^11^ ^]^ acoustics,^[^ ^12^ ^]^ thermal management,^[^ ^13^ ^]^ and mechanical engineering.^[^ ^14^ ^]^ Among these, mechanical metamaterials have garnered particular attention due to their ability to achieve unconventional mechanical behaviors, such as negative Poisson's ratio,^[^ ^15^, ^16^, ^17^ ^]^ programmable stiffness,^[^ ^18^, ^19^, ^20^ ^]^ and energy absorption capabilities.^[^ ^21^, ^22^, ^23^ ^]^ These properties make them highly attractive for applications in flexible electronics,^[^ ^24^ ^]^ soft robotics,^[^ ^25^, ^26^, ^27^ ^]^ aerospace structures,^[^ ^28^, ^29^ ^]^ and biomedical devices.^[^ ^30^ ^]^

The distinctive properties of mechanical metamaterials originate from their capacity to manipulate stress and strain distributions at the microstructural level.^[^ ^31^ ^]^ This enables the design of materials with tailored mechanical responses. For instance, auxetic materials exhibit a negative Poisson's ratio and expand laterally when stretched, resulting in improved energy absorption and fracture resistance.^[^ ^32^, ^33^ ^]^ Similarly, materials with programmable stiffness can adapt their mechanical properties in real‐time, making them ideal for applications in adaptive structures^[^ ^34^, ^35^ ^]^ and soft robotics.^[^ ^36^ ^]^ Despite these advancements, the fixed and singular microstructure of conventional mechanical metamaterials limits their ability to achieve multifunctionality and adaptability. Most existing designs are tailored for specific functions, making it challenging to reconfigure their properties for diverse applications.

This limitation has driven significant interest in the design and development of reconfigurable and programmable metamaterials capable of adapting to diverse operational conditions. Origami,^[^ ^37^, ^38^ ^]^ has emerged as a powerful design framework for engineering materials with complex geometries and tunable properties. Recent advances in origami‐based designs have demonstrated the potential to create metamaterials with unprecedented functionality and adaptability.^[^ ^39^, ^40^, ^41^ ^]^ In parallel, developments in thick‐panel origami structures have further enhanced the practicality of deployable systems in industrial applications by eliminating surface grooves.^[^ ^42^ ^]^ Origami‐inspired metamaterials leverage the principles of folding and bifurcation to achieve a wide range of mechanical behaviors, including tunable stiffness,^[^ ^43^, ^44^, ^45^ ^]^ multistability,^[^ ^46^ ^]^ and programmable Poisson's ratio.^[^ ^43^, ^44^, ^45^, ^46^, ^47^, ^48^ ^]^ By exploiting the geometric transformations inherent in origami patterns, researchers have developed materials that can transition between multiple stable configurations, each exhibiting distinct mechanical properties.^[^ ^49^, ^50^ ^]^ The programmable nature of these origami‐based modules allows for the creation of a metamaterial network with customizable mechanical properties.^[^ ^40^, ^51^ ^]^ This approach not only overcomes the limitations of single‐function designs but also enables the development of multifunctional metamaterials from a single structural framework. For example, the integration of modules with varying stiffness and Poisson's ratio enables the design of materials that simultaneously exhibit energy absorption and vibration damping characteristics.

In this paper, starting from the underlying topology of the MOFs network structure, we present a comprehensive exploration of the design, fabrication, and characterization of reconfigurable origami‐based metamaterials. By leveraging geometric principles and modular assembly techniques, a systematic investigation is conducted on how folding patterns and material properties influence mechanical behavior. Through a combination of experimental validation, computational modeling, and theoretical analysis, the feasibility of achieving programmable Poisson's ratio and variable stiffness properties is demonstrated, enabling precise control over deformation modes and load‐bearing capacity. Furthermore, an in‐depth analysis is conducted on the dynamic response of these structures under cyclic loading, demonstrating their potential for energy absorption and shape‐morphing applications. The academic significance of this work lies in its contribution of a fresh design strategy for origami‐inspired mechanisms, while also offering an alternative perspective on how microstructural architectures can govern the macroscopic properties of materials. At the same time, the high energy absorption and shear resistance brought by the characteristics of zero stiffness/ variable Poisson's have given rise to numerous potential practical application scenarios. For example, in the aerospace industry, lightweight and high‐strength armor made of negative Poisson's ratio structures typically possesses both lightweight and high‐strength characteristics. When impacted by bullets or shrapnel, the material will contract toward the impact point, forming a local high‐density zone, greatly improving its resistance to penetration. Its excellent energy absorption performance can also effectively protect personnel and equipment. In daily consumer goods, metamaterials with variable stiffness can be used in mattresses and sofas to meet the personalized needs of different users by precisely adjusting the “softness and hardness” of the materials. Metamaterials with variable mechanical properties, as a model for new material design, are moving from the laboratory to practical applications, and are expected to bring revolutionary changes in many fields in the future.

## 2 Results and Discussion

### 2.1 Polyhedral Folding

**Figure 1:**
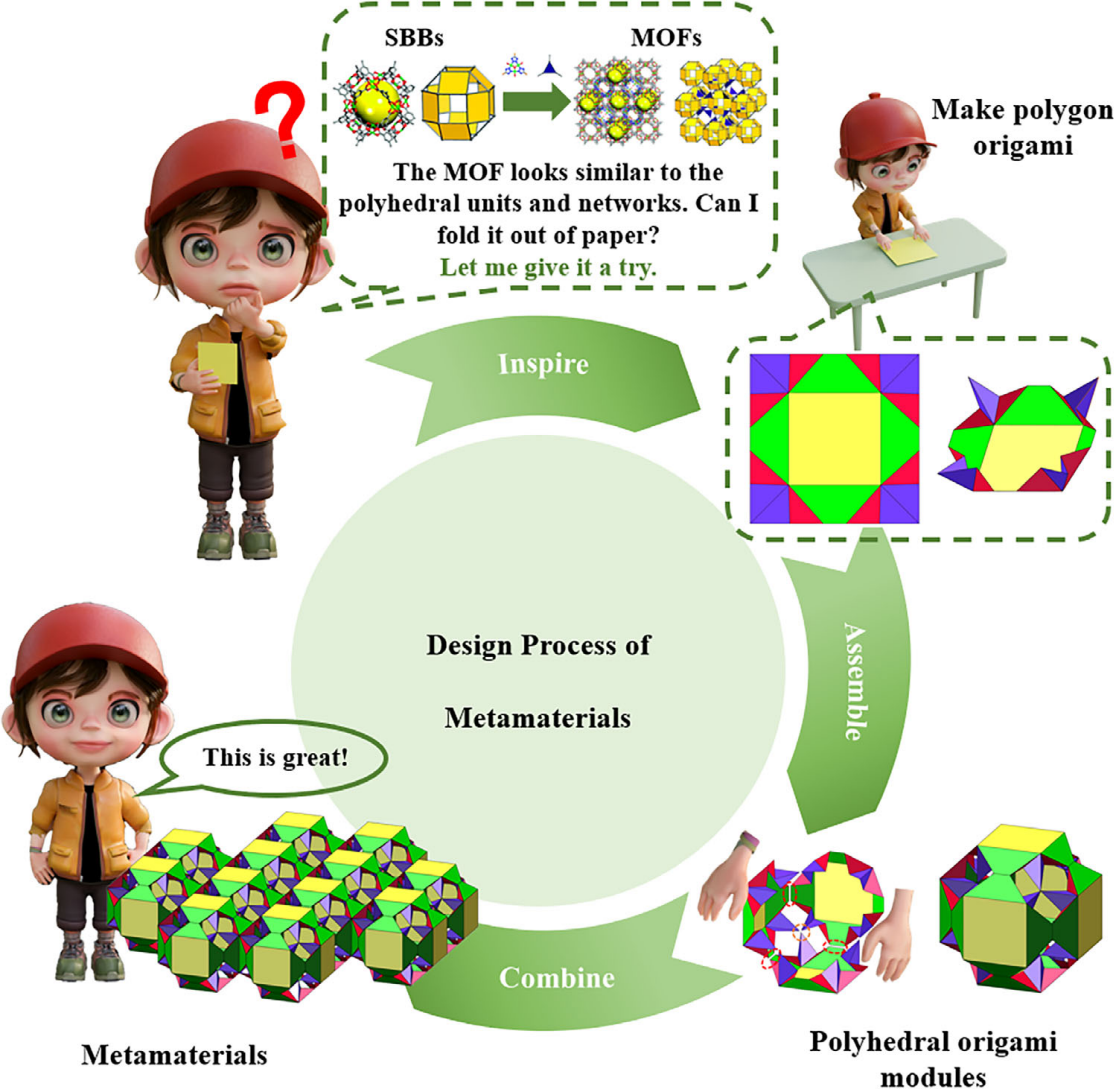
Fabrication process of metamaterials.

Reticle synthesis assembles rigid molecular building blocks designed with precision into ordered networks. These networks have predetermined arrangements and are stabilized by strong bonds. In systematic MOFs chemistry, the key is to combine a specific metal‐containing secondary building unit (SBU) with various organic SBUs. The structure is then extended in space according to the crystal symmetry.^[^ ^52^ ^]^ The use of externally functionalized metal‐organic polyhedra as supramolecular building blocks to construct unique networks with high connectivity is a strategy already established in MOF design and synthesis.^[^ ^53^, ^54^ ^]^ For example, in **Figure** **1**, in situ generated truncated cubic octahedral supramolecular building blocks^[^ ^54^ ^]^ (SBB) are often used as tertiary structural units to assemble the overall MOF structure. In this context, the origami design proposed in this paper can be directly viewed as a physical simulation of the cubic octahedral SBB polyhedral hierarchy in this type of MOF. Specifically, the resulting rhombic dodecahedron corresponds to the SBBs used in the MOF. All the green trapezoidal planes in the crease pattern represent stable secondary building blocks—such as copper paddlewheel clusters—while the 24 intersecting nodes of the rod‐like structures correspond to the 1,3‐BDC linking groups connecting the metal clusters, thus ensuring that the bending angle of the bridging ligands is exactly 120°. This precise correspondence from molecular topology to macroscopic geometry successfully transforms complex MOF structures into intuitive origami models, thus providing a clear blueprint for the design and understanding of metamaterials with specific mechanical properties (such as porosity and deformability). Thus, we designed a polyhedral origami and used it as the basis for metamaterials. The initial step involves designing the crease pattern on the polygonal structure in Figure 1.

To enable foldability, creases are introduced into the square panel and symmetrically applied at the remaining three vertices in an identical configuration. By connecting edges and vertices (including equivalent geometric elements), the polygon origami pattern is copied six times and assembled to obtain a foldable polyhedron. The top and bottom surfaces of the polyhedral structure are interconnected through four distinct branches. Constraints also exist on the connectivity between vertices located on different structural branches of the polyhedron.

**Figure 2:**
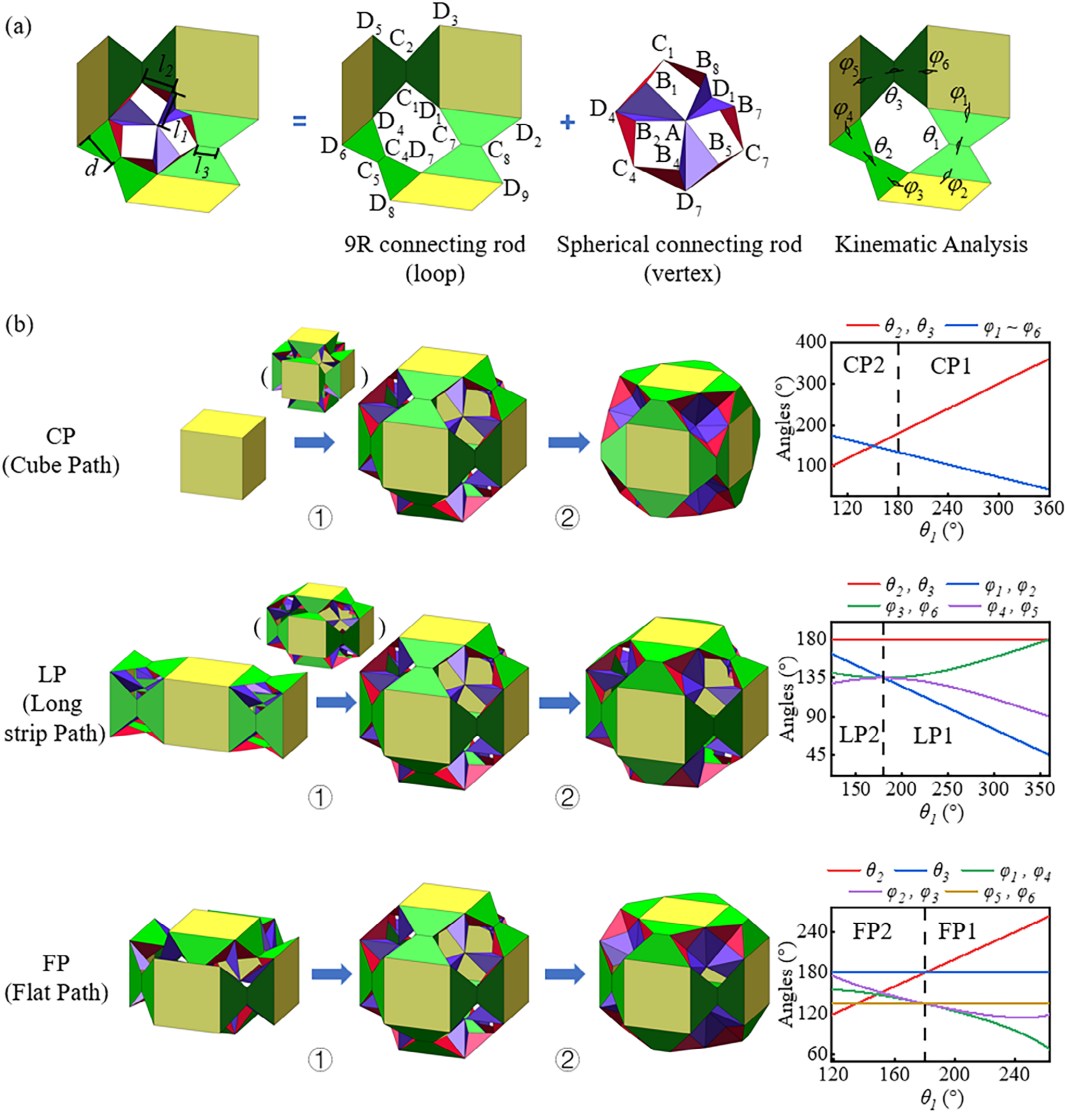
Polyhedral units. a) Kinematic modeling of polyhedral origami. b) Fork paths and kinematic relationships.

The smallest loop is extracted from the polyhedral origami, encompassing three faces and one vertex in **Figure** **2**a. The motion of this loop has been proven to represent the motion of the entire polyhedron. The loop pattern can be divided into two parts: the 9R loop and the point connected loop. Motion analysis (Section S1, Supporting Information) reveals that the polyhedral origami exhibits multiple degrees of freedom, primarily due to the 9R loop structure. In contrast, point‐connected loops do not introduce local mobility. Therefore, when polyhedral origami is used as a module combination, constraints need be added to transform it into a single degree of freedom origami module. The polyhedral origami structure exhibits three degrees of freedom in Equation S1‐3 (Supporting Information). The addition of two constraints to the 9R loop eliminates two degrees of freedom, resulting in the point‐connected loop's motion being uniquely constrained by the 9R loop. Based on this, three methods of adding constraints have been derived (Figure S1‐1, Supporting Information), namely adding: center symmetry constraint (Figure S1‐1‐a, Supporting Information), one face symmetry constraint and fixing one crease (Figure S1‐1‐b, Supporting Information), and fixing two creases (Figure S1‐1‐c, Supporting Information). From this, polyhedral origami generates three types of single degree of freedom metamaterial modules (as shown in Figure 2b), corresponding to three different branches of motion: cube path (CP), long strip path (LP), and flat path (FP).

Equations S1‐4–6 (Supporting Information) show the motion relationships of three motion branches, corresponding to the motion of each polyhedron in Figure 2b. The CP module maintains the symmetry of the cube during motion, while the θ1,  θ2,  θ3, and φ1,  φ2,  φ3,  φ4,  φ5,  φ6, have the same variation. When *θ*_1_ increases, it is denoted as CP1. At θ1  =  360°, the CP module reaches its minimum volume. When θ1 is reduced, it is recorded as CP2. At θ1  =  100.63°, the three sets of red and purple triangles at the vertices of the polyhedron reach a coplanar state, corresponding to the right‐hand state of the CP module. Similarly, during the motion of the LP module, only the side surfaces maintain the symmetry of a square, with φ1  =  φ2,  φ4  =  φ5,  φ3  =  φ6. When θ1  =  360°, the LP1 module has a maximum lateral distance of 129.6 mm. When the two sets of red and purple triangles at the vertices of the polyhedron reach a coplanar state, corresponding to the right‐hand state of the LP module, LP2 module reaches the end point of motion. During the movement of the FP module, only the upper and lower surfaces maintain square symmetry, with θ1  =  θ2, φ1  =  φ4, φ2  =  φ3. When θ1  =  262.78°, the two sets of red and purple triangles at the vertices of the polyhedron reach a coplanar state, corresponding to the right‐hand state of the FP module, the FP1 module reaches the endpoint of motion. When θ1  =  118.39°, the red triangles and green trapezoids of the polyhedron reach a coplanar state, corresponding to the left state of the FP module, the FP2 module reaches the endpoint of motion. Figure S1‐2 (Supporting Information) shows the variation of geometric dimensions of three motion branches with θ1. For the CP module, the dimensions of length, width, and height are equal, initially increasing and subsequently decreasing as θ1 increases. The LP module shows a different pattern, where the length decreases first and then increases. Meanwhile the height and width change in the same way as the CP module. For the FP module, the height initially reaches a peak and subsequently decreases but the length and width stay unchanged. The three motion branches of CP, LP, and FP share a common shape, which corresponds to the bifurcation point of polyhedral origami motion. For a certain branch of motion, at this point, the fold where θ1,  θ2,  θ3 is located in polyhedral origami can be folded inward or outward, forming six motion paths CP1, CP2, LP1, LP2, FP1, FP2. According to the constraint setting method, only consider the situation where the side panel folds inward or outward relative to the polyhedral module at the same time.

### 2.2 Reconfigurable Origami Metamaterials

**Figure 3:**
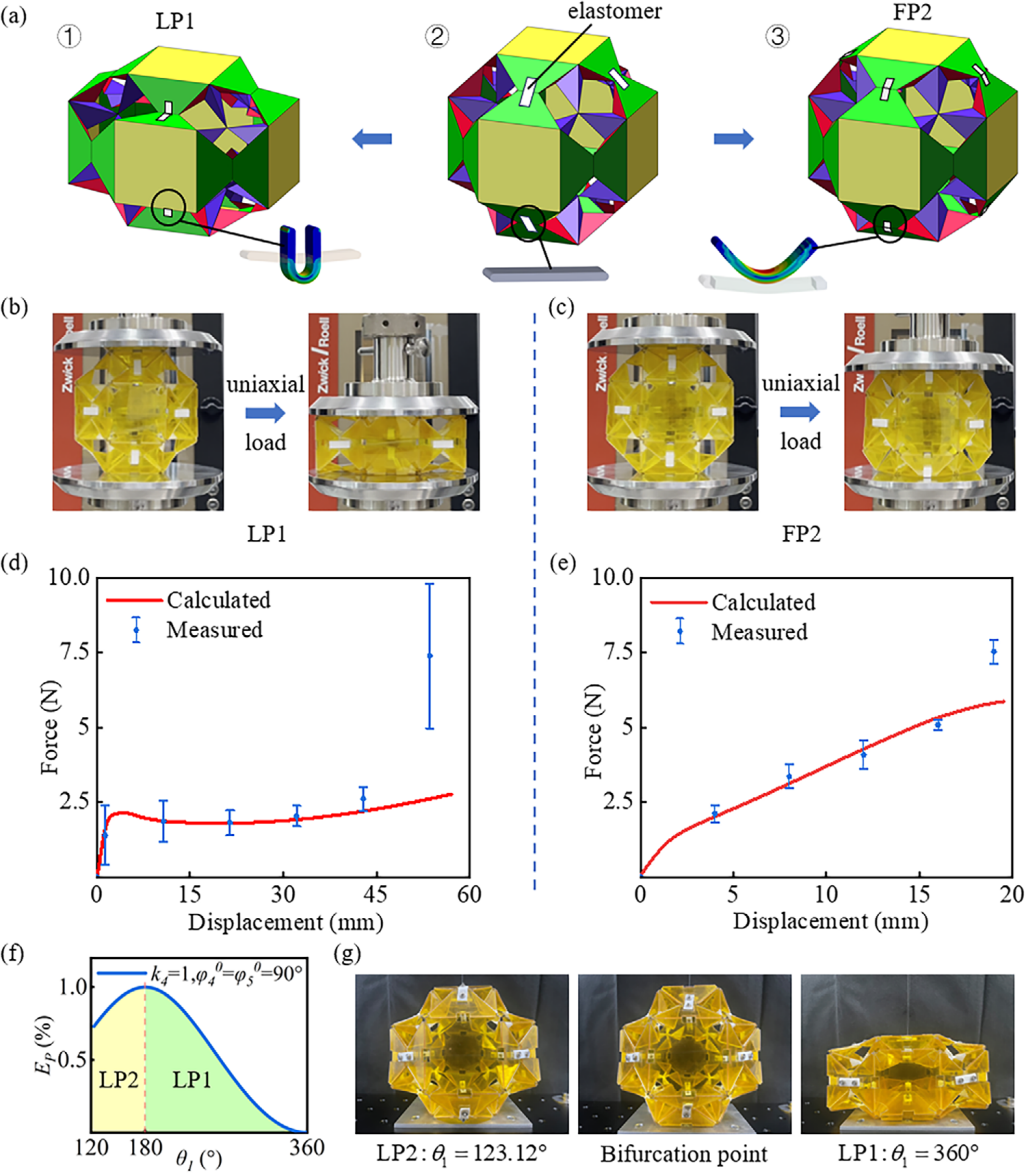
Coupled rigid‐flexible design of Polyhedral units. a) Rigid flexible coupling design with added elastomer. b,c) Uniaxial loading experiment of the metamaterial. d,e) Theoretical and experimental values of force displacement under uniaxial load. f,g) Bistable implementation of the metamaterial module.

After completing the design and kinematic analysis of polyhedral origami, the variable stiffness characteristics of each module, acting as a metamaterial unit cell, can be elucidated through rigid‐flexible coupled design. An elastic joint utilizing rigid origami can be obtained after adding an elastic body at the crease (θ1,  θ2,  θ3 and their equivalent positions) in **Figure** **3**a‐2. After adding the three constraints in Figure S1‐1 (Supporting Information), the origami polyhedron becomes a single degree of freedom system. Therefore, the reliable movement of creases largely depends on the reliability of the constraints added. As shown in Figure 3a, the current symmetric constraint is formed by connecting the same elastic body at the joint where the constraint is located. Figure 3a‐1–3 illustrate the different elastic addition strategies adopted for metamaterial modules with different bifurcation paths (LP1, FP2). According to the kinematic relationship of polyhedral origami (Equation S1‐5–6, Supporting Information), diverse deformation characteristics are observed in the elastic body upon initiating from the bifurcation point. Boundary conditions are established in accordance with the addition method and motion scenario in Figure 3a‐1,3. Using TPU‐95A as the material, a finite element simulation of the elastic joint is conducted. Due to the low velocity of the uniaxial loading experiment, the process is considered quasi‐static. The geometric angle relationship within the kinematic models (Equation S1‐5–6, Supporting Information) of the LP and FP modules provides a foundation for analyzing deformation. Elastic force arises during the deformation process. By applying the static equilibrium equation (LP1 in Section S2, Supporting Information), this force is translated into the longitudinal uniaxial load necessary for polyhedral folding.

As a result, the force‐displacement curves of two metamaterial modules (LP1 and FP2) under uniaxial loading are obtained through theoretical analysis (red lines in Figure 3d,e). The mechanical testing of the metamaterial module under uniaxial load, depicted in Figure 3b,c, indicating that the mechanical response characteristics and trends align between theoretical and experimental results. The LP1 module exhibits a longer axial displacement (≈58 mm), which is governed by its bifurcation path in Figure 3d,e. Meanwhile, in the middle and early stages of the motion (with a longitudinal strain less than 0.5), the applied uniaxial load is basically maintained at 2.5N, which exhibits the characteristic of quasi zero stiffness, indicating that the LP1 module has good shock absorption and buffering effects. The FP2 module exhibits a shorter axial movement distance (≈19 mm), and its force displacement curve maintains a linear increase throughout the motion. Compared to the LP1 module, the FP2 module requires a larger uniaxial load under the same longitudinal strain. In the latter stage of the uniaxial loading experiment, as deformation increases further, both types of modules are displaced to their limit positions to form a structured structure. This also leads to a sharp increase in the measured load value at the end of the curve, causing a significant deviation from the theoretical value.

Multiple stable states can be engineered by modifying the initial geometry and configuration of the elastomer. Taking the LP module as an example, its motion follows two bifurcation paths: LP1 and LP2. According to the motion relationship in the LP path in Figure 2b, both φ4 and φ5 show a rising trend followed by a decline as θ1, increases. The elastic arrangement at θ1,  θ2,  θ3 is eliminated and substituted with a thin flexible connector based on this trend. Elastic arrangements are introduced at the creases located at φ4 and φ5. The initial angle of the elastic arrangement is φ4°  =  φ5°  =  90°, resulting in a folding module with bistable properties. Due to the deformation of TPU elastomer within the linear elastic range, the simplified model of elastic potential energy based on Hooke's law (Figure 3f) shows that the maximum position of its elastic potential energy happens to be at the bifurcation point. The lowest point of elastic potential energy is at LP1: θ1  =  360°, LP2: θ1  =  123.12° in Figure 3g. This corresponds to the two steady‐state positions of the LP module. However, due to interference issues, there was a slight deviation in the shape of the module in the experiment.

Based on the different lateral size responses of three metamaterial modules under the same longitudinal displacement, metamaterials also have the characteristic of variable Poisson's ratio. According to the kinematic relationship, Equation 1 shows the geometric dimensions of the cell in the X, Y, and Z orthogonal directions.

(1) $$\left\{\begin{array}{c} L=2l_{1}+l_{2}+l_{3}+2d_{0}\left(\sin\varphi_{3}-\cos\varphi_{4}-1\right)\\ D=2l_{1}+l_{2}+l_{3}+2d_{0}\left(\sin\varphi_{1}-\cos\varphi_{2}-1\right)\\ H=2l_{1}+l_{2}+l_{3}+2d_{0}\left(\sin\varphi_{5}-\cos\varphi_{6}-1\right) \end{array}\right.$$

According to the definition of Poisson's ratio:

(2) $$v_{ZX}=-\frac{\varepsilon_{L}}{\varepsilon_{H}},\;v_{ZY}=-\frac{\varepsilon_{D}}{\varepsilon_{H}}$$

Considering that the Poisson's ratio of metamaterial modules does not necessarily remain constant as strain increases, the above equation has a differential form:

(3) $$v_{ZX}=-\;\frac{\varepsilon_{L}}{\varepsilon_{H}}=-\frac{dL\cdot H}{dH\cdot L},\;v_{ZY}=-\;\frac{\varepsilon_{D}}{\varepsilon_{H}}=-\frac{dD\cdot H}{dH\cdot D}$$

**Figure 4:**
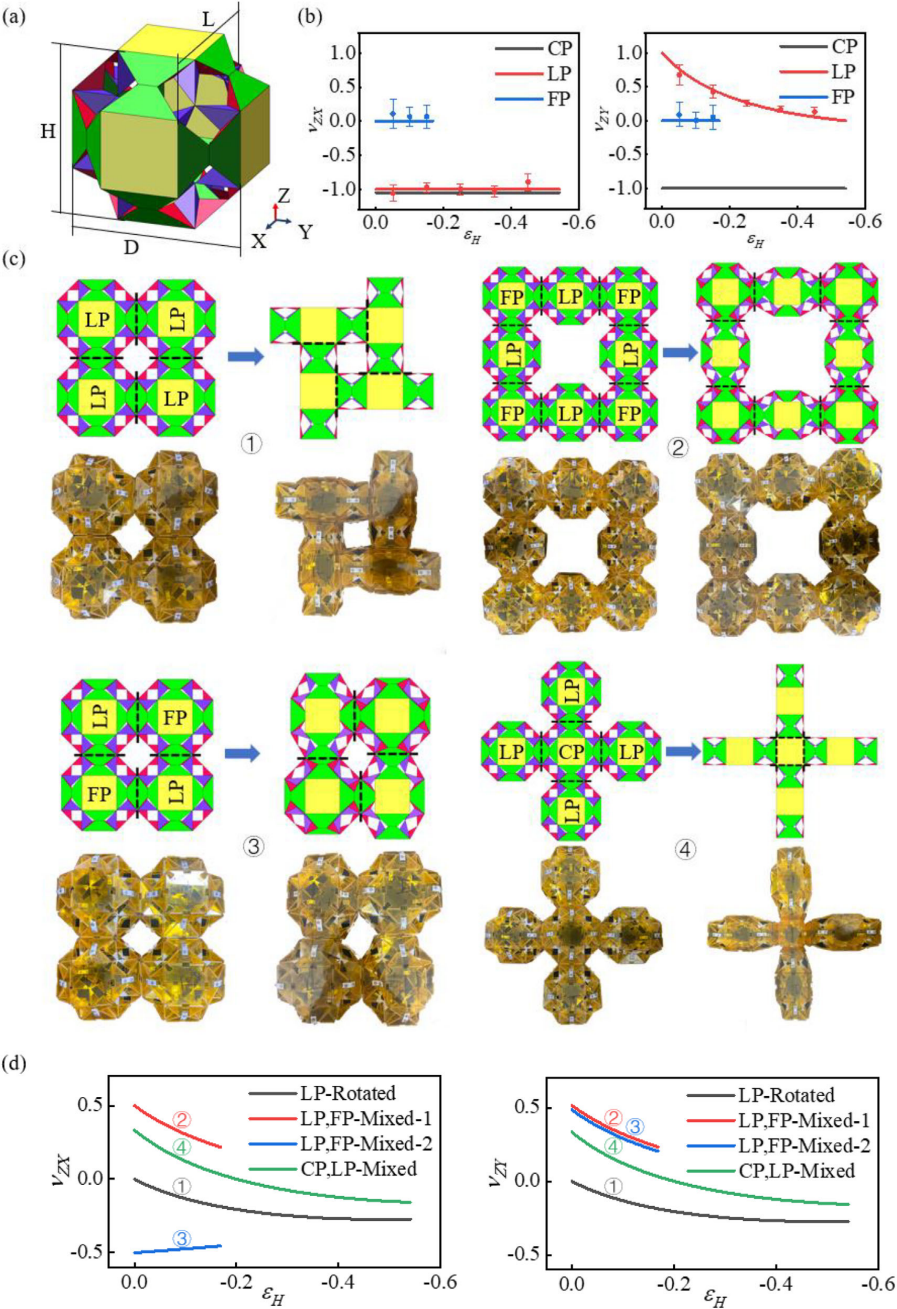
Poisson's ratio of metamaterial modules. a) Geometric Definition of Metamaterial Modules. b) Poisson's ratio of a single metamaterial module. c) Different networking methods between modules: ①LP‐Rotated ② LP, FP‐Mixed‐1 ③ LP, FP‐Mixed‐2 ④ CP, LP‐Mixed. d) Longitudinal strain Poisson ratio relationship of each network.

The Poisson's ratios of the three metamaterial modules CP, LP, and FP can be obtained from Equation 3, and their Poisson's ratios in ν_ZX_ and ν_ZY_ directions are shown in **Figure** **4**b. For the CP module, the Poisson's ratios in the orthogonal X and Y directions consistently equal −1, implying that the relative contractions in L and D directions match the contraction of H in magnitude. On the contrary, the two Poisson's ratios of the FP module consistently remain 0, signifying that the lateral dimensions L and D do not change as the longitudinal strain increases. Compared with the former, for the LP module the Poisson's ratios in νZX and νZY directions are different. The degree of contraction in L is equivalent to that in H, and the Poisson's ratio in νZX direction remains constant −1. D shows an increasing trend during the contraction of H, resulting the Poisson's ratio in νZY direction ≥ 0.

The CP, LP, and FP metamaterial modules share the same structural configuration at the bifurcation point, enabling them to form networks through different connection patterns. To avoid singular situations at bifurcation points, CP1 and LP1, which exhibit larger longitudinal strains, are selected to represent the CP and LP modules. Meanwhile, FP2, with better support capacity, is chosen to represent the FP module. Figure 4c presents four networking methods, illustrated via a top view for clarity: LP's own rotating networking (LP‐Rotated); LP and FP hybrid networking‐1 (LP, FP‐Mixed‐1), LP and FP hybrid networking‐2 (LP, FP‐Mixed‐2), CP and LP hybrid networking (CP, LP‐Mixed). The metamaterials after networking can still be used as modules to continue the array, and Equation (3) remains applicable to these modules. Figure 4d shows the calculation results of Poisson's ratio, from which positive Poisson's ratio (LP, FP‐Mixed‐2), zero Poisson's ratio (FP2), negative Poisson's ratio (CP, LP‐Mixed), and various metamaterial modules with distinct properties can be identified.

**Figure 5:**
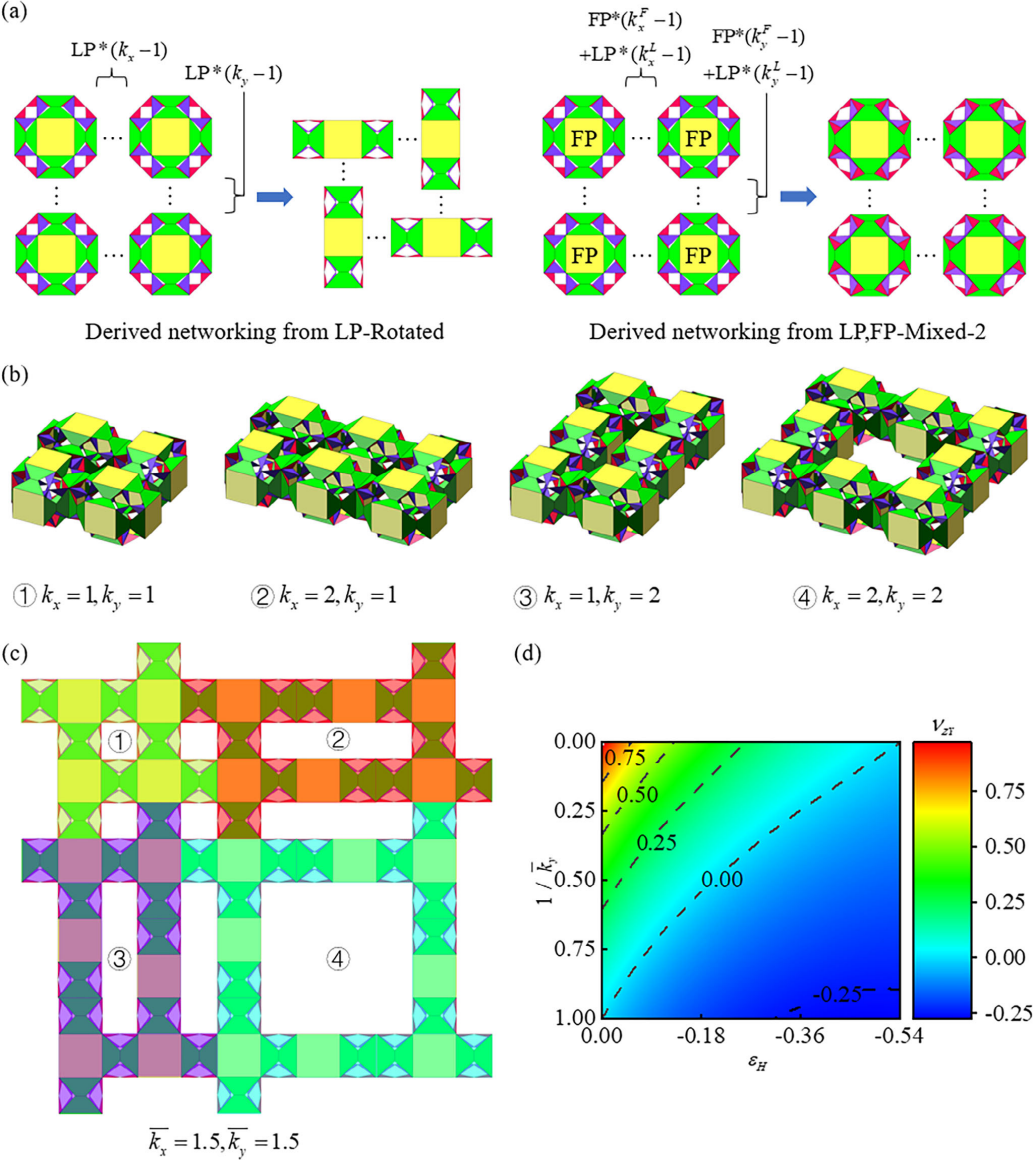
Metamaterial‐Derived networks and programmable Poisson's Ratio. a) LP module is incorporated into the LP‐Rotated network. LP and FP modules are added to the LP, FP‐Mixed‐1 network. b) Four distinct derivative networks derived from the LP‐Rotated network. c) Recombination of derivative networks derived from LP‐Rotated network, including ①*k*_x_ = 1,*k*_y_ = 1,②*k*_x_ = 2,*k*_y_ = 1,③*k*_x_ = 1,*k*_y_ = 2,④*k*_x_ = 2,*k*_y_ = 2. d) Poisson's ratio achievable by recombined metamaterials in derived networking from LP‐Rotated.

Based on several networking methods shown in Figure 4c, by changing certain basic modules in the networking, more diverse Poisson's ratio networks can be created. In Figure 4d, it can be preliminarily seen that the Poisson's ratio of the metamaterial network depends on the type, quantity, and arrangement of the basic modules that make up it. This provides a method and theoretical support for arbitrarily converting Poisson's ratio within a specified range and achieving programmable Poisson's ratio. We attempted this idea in **Figure** **5**a, taking the LP rotation network as an example, by adding any number of long side LP modules along the X or Y direction. The added section in the X or Y direction consists of (*k*x or *k*y) long‐edge LP modules. The integration methods of LP and FP modules into the LP, FP‐Mixed‐1, and LP, FP‐Mixed‐2 networks are illustrated in the right panel of Figures 5a and S3‐1 (Supporting Information). The incorporation of LP modules into the CP, LP‐Mixed networks is also presented.

Figure 5b shows several derivative networks of LP‐Rotated networking with the addition of basic modules. Equation S3‐1 (Supporting Information) presents their Poisson's ratio. νZX depends solely on *k*x and νZY is exclusively determined by *k*y, which means the Poisson's ratio in two orthogonal directions can be independently controlled. Taking *ν*_ZX_ as an example, under the same strain, νZX of the derivative network increases with the increase of *k*x; When the value of *k*x is determined, νZX of the derivative network decreases as the longitudinal strain εH increases; When *k*x  =  1, νZX is the same as the LP‐Rotated network; When *k*x → +∞, ν_ZX_ is the same as νZY of the LP module.

A single derivative network can form metamaterials in an array, while different derivative networks can also be combined into metamaterials. In Figure 5c, basic module 1 (*k*x  =  1,*k*y  =  1) can form a network of $\bar{k}$_x_ =  1.5, $\bar{k}$_y_ =  1.5 with derivative modules 2 (*k*x  =  2,*k*y  =  1), 3 (*k*x  =  1,*k*y  =  2), and 4 (*k*x  =  2,*k*y  =  2). Moreover, there are multiple ways to combine a specific method in Figure S3‐1‐a (Supporting Information). The network composed of four rotated derivative modules 2 (*k*x  =  2,*k*y  =  1)has the same Poisson's ratio as the recombined network ($\bar{k}$_x_  =  1.5, $\bar{k}$_y_  =  1.5) in Figure 5c. The recombination of derivative networks broadens the range of achievable Poisson's ratios in metamaterials in Equation S3‐2 (Supporting Information). Consequently, *k*x and *k*y are no longer constrained to positive integers. The Poisson's ratio ν$v\in \left(-0.274,1\right)$ can be achieved by combining LP‐Rotated derived networks in Figure 4d. In addition, the networking methods in Figure 3b have many derivatives that can be further combined. Figure S3‐3–5 (Supporting Information) illustrate the derivative networks of LP, FP‐Mixed‐1, LP, FP‐Mixed‐2, and CP, LP‐Mixed configurations. These networks can be recombined to form more diverse metamaterials. Figure S3‐6–8 (Supporting Information) demonstrate the attainable Poisson's ratios of these three metamaterials. Through this combination, metamaterials are programmable to possess diverse variation curves within the range of ν$v\in \left(-0.5,1\right)$.

### 2.3 Production and Application

**Figure 6:**
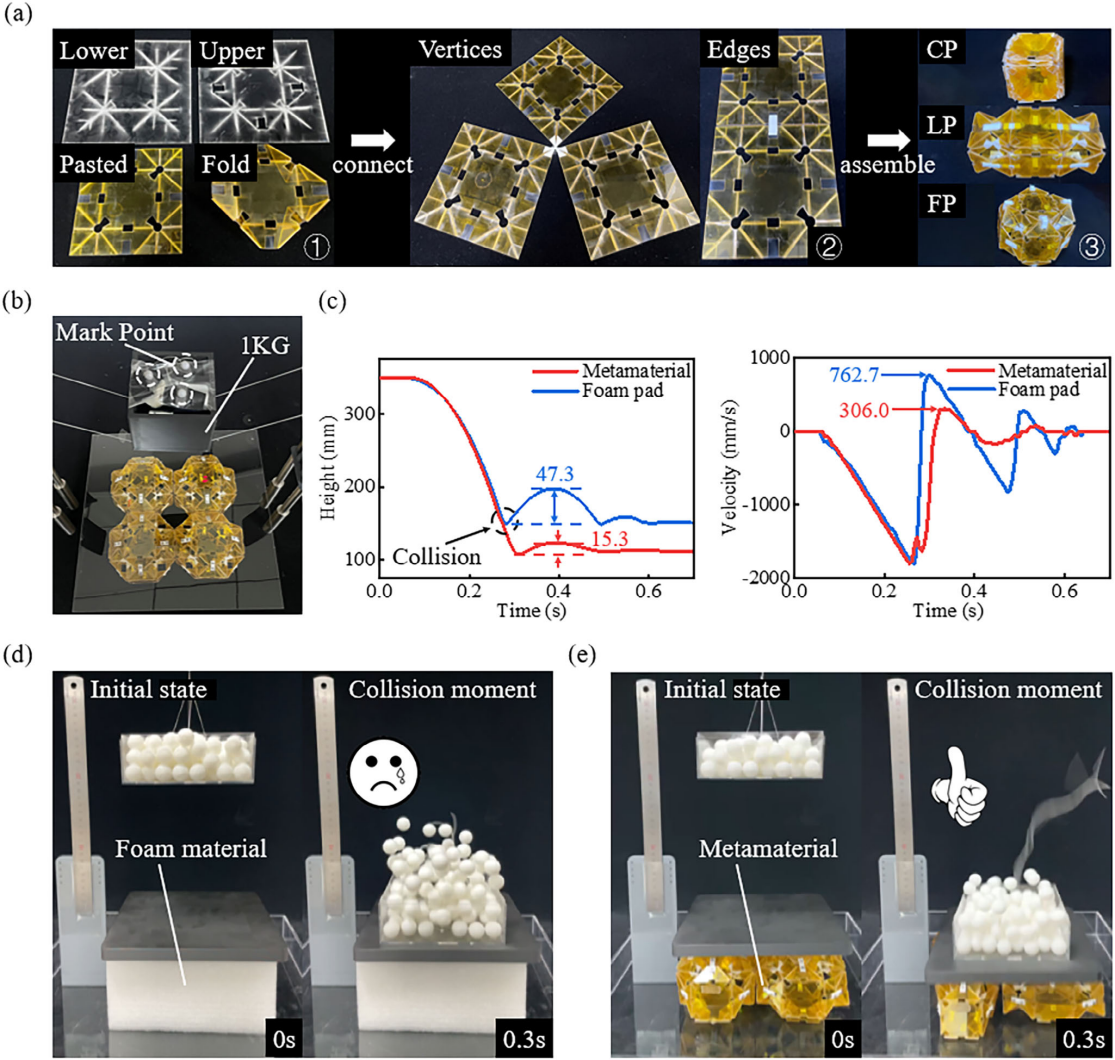
Experimental Comparison of Metamaterial Shock Absorption Performance. a) Manufacturing process of the metamaterial module. b,c) 1 kg weight drop impact test. d,e) Ball shock absorption experiment.

To eliminate the influence of fragile creases and enhance the rigidity of the overall model, double‐layer acrylic sheets are employed to fabricate the folding surface. High‐temperature resistant double‐sided tape is applied at the creases to simulate fold lines in Figure S4‐1 (Supporting Information). Laser cutting is employed to remove excess material, enabling the folding mechanism to exhibit unidirectional folding behavior. **Figure** **6**a‐1 shows the manufacturing process of the polygonal origami mechanism, corresponding to the upper acrylic cutting pattern (Figure S4‐2‐a, Supporting Information), the lower acrylic cutting pattern (Figure S4‐2‐b, Supporting Information), the overall cutting pattern after pasting (Figure S4‐2‐c, Supporting Information), and the polygonal origami subsequent to removal of excess materials. Figure 6a‐2 details the process of making polyhedral origami by connecting points and faces in polygonal origami, and Figure 6a‐3 exhibits metamaterial modules (CP: θ1  =  360°, LP: θ1  =  360°, FP: θ1  =  118.39°) in different forms.

Characterized by quasi‐zero stiffness and negative Poisson's ratio, metamaterials offer superior shock mitigation and buffering performance. An impact experiment is conducted in Figure 6b, where a 1‐kg weight fell freely from a height of 200 mm, and the motion data of the Mark Point is recorded by tracking. In Figure 6c, the maximum bounce height from the lowest point of the heavy object reaches 15.3 mm, and the maximum bounce velocity is 306 mm s^−1^ when the LP‐Rotated network metamaterial is used as a shock absorber pad. Conversely, the use of foam damping materials results in a maximum rebound height of 47.3 mm for the weight, accompanied by a maximum rebound velocity of 762.7 mm s^−1^. Compared with foam shock absorption materials, metamaterials have lower bounce height and slower maximum bounce speed, which have better shock absorption and energy absorption effects.

To better demonstrate this feature, a transparent acrylic cubic container with dimensions of 150 mm × 150 mm is filled with 3D‐printed spheres, each with a diameter of 18 mm. The total weight of the box and ball is ≈300 g, and the suspension height is 200 mm. The thin rope is sectioned to allow the square box to fall freely. In the drop tests of small spheres, the use of metamaterials as shock absorbers resulted in a total of 6 sphere ejections over 10 repeated trials. In contrast, the use of foam materials as shock absorbers resulted in the ejection of 76 balls during the experiments. These results indicate that metamaterials exhibit superior shock absorption performance compared to foam materials under the tested conditions.

The impact moment of the ball is compared within the 0.3 s time window highlighted in Figure 6d,e. The maximum bounce height of the ball under the influence of the metamaterial shock pad is significantly lower than that observed with the foam material. These effects primarily result from two factors: the substantial longitudinal deformation capability of the metamaterials and their characteristic zero stiffness during motion. As shown by the blue line in Figure 6c, traditional elastic materials undergo a process of gradually decreasing oscillation velocity when subjected to large impacts, resulting in a significant reverse velocity of the object at the moment of collision. This is due to the characteristic of their positive stiffness (increasing elastic force with increasing deformation). On the contrary, in the red line of Figure 6c, we can see that LP metamaterials have the characteristic of quasi zero stiffness (with increasing deformation, the elastic force remains basically unchanged), resulting in a decrease in the oscillation period of slow velocity and a low peak value, which makes the rebound speed of the object relatively low. In the experiments shown in Figure 6d,e, the number of balls ejected also corresponds to this difference. At the same time, the metamaterial network of LP Rotated also has a certain negative Poisson's ratio characteristic, which increases its ability to absorb shock and energy.

## 3 Conclusion

In this paper, drawing inspiration from network chemistry and the 3D crystalline frameworks of MOFs, an effective reconfigurable polyhedral origami pattern has been designed. After incorporating different constraints, three different single degree of freedom (CP, LP, FP) patterns emerged as the three basic modules of metamaterials. Elastomers are integrated into the metamaterial modules to enhance their mechanical behavior. Subsequent uniaxial compression tests and simulations reveal that two of the basic modules exhibited quasi‐zero stiffness and positive stiffness respectively. Meanwhile, the modules are found to exhibit three types of Poisson's ratios: zero, −1, and positive values. By combining and arranging these modules, programmable metamaterials with any Poisson's ratio ranging from −1 to 1 have been achieved. With practical application in mind, a shock absorption experiment is developed based on these two special mechanical properties of the metamaterial. The effectiveness of its shock absorption is confirmed, demonstrating strong potential for practical engineering use.

Overall, this study applies reconfigurable polyhedral origami to the design of mechanical metamaterials, achieving unconventional mechanical performance of mechanical metamaterials and affording better flexibility in their mechanical properties. This approach offers groundbreaking applications in emerging domains such as programmable quantum waveguides for topological photonics, phase‐transitional armor with dynamic stiffness modulation, and biohybrid metasurfaces for neural interface engineering. This signifies a transformative advancement in designer materials, enabling on‐demand multifunctionality that transcends natural limitations.

## 4 Experimental Section

### Metamaterial Model Making

In the manufacturing process, polymethyl methacrylate (PMMA) with a thickness of 0.5 mm was selected as the acrylic sheet material. Polyimide tape was employed as the high‐temperature‐resistant double‐sided adhesive. The precision laser‐cutting equipment employed was the I LASER3000 model. The elastic connector was fabricated via 3D printing, with Bambu‐TPU95A as the printing material. The rigid body connector was produced following the same procedure, utilizing Bambu‐PLA‐Basic as the consumable material. The 3D printer model was Bambu‐X1‐Carbon, and instant dry adhesive was used to connect the acrylic board and the elastic/rigid body.

### Experimental Setup

Single axis loading test was conducted using a material mechanics testing machine (model: BT2‐FA010TH. A50.002) with a loading rate of 0.01 mm s^−1^ and a loading end condition of 10N. Using a motion capture system (Nokov optical 3D motion capture system with measurement error less than 0.01 mm) to measure the lateral and longitudinal displacement, velocity, and acceleration of various surfaces of metamaterials.

## Conflict of Interest

The authors declare no conflict of interest.

## Supporting information



Supporting Information

Supplemental Video 1

Supplemental Video 1

## Data Availability

The data that support the findings of this study are available in the supplementary material of this article.
