# Supplementary material for: 3D‐MOF‐Lattice Inspired Programmable Metamaterials Based on Reconfigurable Polyhedral Origami
Source: Adv Sci (Weinh). 2025 Dec 14;13(9):e17921. doi: 10.1002/advs.202517921 (PMC12904030; doi:10.1002/advs.202517921)
Supplement: Supplementary file 1 — Supporting Information [file ADVS-13-e17921-s002.pdf]

Supporting information

3D-MOF-Lattice Inspired Programmable Metamaterials based on Reconfigurable Polyhedral Origami

Xi Kang[#], Yangqin Zhang[#], Hongshuang Fan, Ziyan Xu, Yue Dong, Bing Li**

X. Kang, Y. Dong, B. Li

Guangdong Provincial Key Laboratory of Intelligent Morphing Mechanisms and Adaptive Robots, Harbin Institute of Technology, Shenzhen, 518055, China

E-mail: dongyue@hit.edu.cn (Yue Dong); libing.sgs@hit.edu.cn (Bing Li)

X. Kang, Y. Zhang, H. Fan, Z. Xu, Y. Dong, B. Li

School of Robotics and Advanced Manufacture, Harbin Institute of Technology, Shenzhen, 518055, China

The supporting information include:

S1. Kinematic analysis of polyhedral origami

S2. Static analysis of polyhedral origami

S3. Poisson's ratio calculation for networking

S4. Fold design of polyhedral origami

S1. Kinematic analysis of polyhedral origami

S1.1 Geometric modeling

As shown in **Figure 2-a** from the main text, extract the minimum loop analysis from the polyhedron and divide it into two parts for solving.

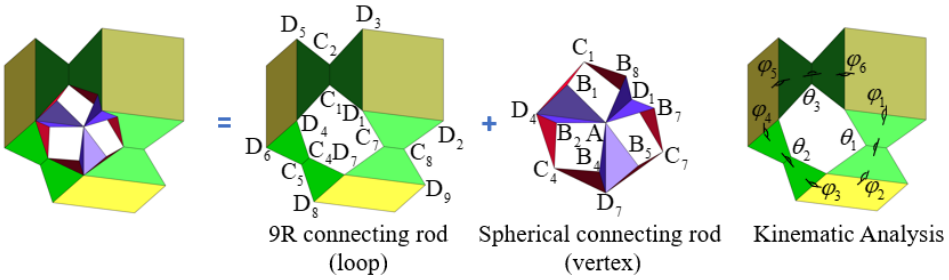

Figure 2-(a). Kinematic modeling of polyhedral origami.

Firstly, during the process of polyhedral motion, the 9R loop always has geometric conditions:

$$\begin{cases} D_1D_2 \,//\, C_7C_8 \,//\, D_7D_9 \\ D_4D_5 \,//\, C_1C_2 \,//\, D_1D_3 \\ D_7D_8 \,//\, C_4C_5 \,//\, D_4D_6 \\ D_1D_2 \perp D_1D_3 \\ D_4D_5 \perp D_4D_6 \\ D_7D_8 \perp D_7D_9 \end{cases} \tag{S1-1}$$

Under these geometric constraints, three planes $D_1D_2D_3$, $D_4D_5D_6$ and $D_7D_8D_9$ remain mutually perpendicular. Lines D_1D_2 and D_4D_6 lie in the same plane, as do lines D_1D_3 and D_7D_8, and lines D_4D_5 and D_7D_9. Lines D_1D_2 and D_4D_6 are equidistant from the plane $D_7D_8D_9$. Likewise, lines D_7D_9 and D_4D_5 exhibit equal perpendicular distances to the plane $D_1D_2D_3$, and lines D_1D_3 and D_7D_8 are equidistant from the plane $D_4D_5D_6$.

According to the geometric parameters of the 9R loop set in **Figure 2-a**, the following relationships are established:

$$\begin{cases} l_{D_1D_2\sim D_7D_8D_9} = d_0(\sin\varphi_2 - \cos\varphi_1) \\ l_{D_4D_6\sim D_7D_8D_9} = d_0(\sin\varphi_3 - \cos\varphi_4) \\ l_{D_7D_9\sim D_1D_2D_3} = d_0(\sin\varphi_1 - \cos\varphi_2) \\ l_{D_4D_5\sim D_1D_2D_3} = d_0(\sin\varphi_6 - \cos\varphi_5) \\ l_{D_1D_3\sim D_4D_5D_6} = d_0(\sin\varphi_5 - \cos\varphi_6) \\ l_{D_7D_8\sim D_4D_5D_6} = d_0(\sin\varphi_4 - \cos\varphi_3) \\ \varphi_1 + \varphi_2 + \theta_1 = 450^\circ \\ \varphi_3 + \varphi_4 + \theta_2 = 450^\circ \\ \varphi_5 + \varphi_6 + \theta_3 = 450^\circ \end{cases} \tag{S1-2}$$

Based on the geometric relationship outlined above, the following equations hold:

$$\begin{cases} \sin\varphi_4 - \cos\varphi_3 = \sin\varphi_5 - \cos\varphi_6 \\ \sin\varphi_3 - \cos\varphi_4 = \sin\varphi_2 - \cos\varphi_1 \\ \sin\varphi_1 - \cos\varphi_2 = \sin\varphi_6 - \cos\varphi_5 \\ \varphi_1 + \varphi_2 = 450° - \theta_1 \\ \varphi_3 + \varphi_4 = 450° - \theta_2 \\ \varphi_5 + \varphi_6 = 450° - \theta_3 \end{cases} \tag{S1-3}$$

Six independent constraint equations are formulated for the unknowns $\theta_1, \theta_2, \theta_3$ and $\varphi_1, \varphi_2, \varphi_3, \varphi_4, \varphi_5, \varphi_6$, indicating that the 9R loop has multiple degrees of freedom.

Next, we will analyze the point connection loop. According to the above analysis, the 9R loop has multiple degrees of freedom, so the position of point $C_1, C_4, C_7, D_1, D_4, D_7$ is determined by $\theta_1, \theta_2, \theta_3$ and $\varphi_1, \varphi_2, \varphi_3, \varphi_4, \varphi_5, \varphi_6$. Due to the existence of a definite length relationship $AD_1 = AD_4 = AD_7 = \sqrt{2}d$, if the configuration of the 9R loop is uniquely determined, the position of point A is also uniquely determined. When points A, C_i, D_i are determined by $\theta_1, \theta_2, \theta_3$ and $\varphi_1, \varphi_2, \varphi_3, \varphi_4, \varphi_5, \varphi_6$, the position of the point B_i can be determined. These results indicate that the vertex configuration of the polyhedron is governed by the 9R loop, thereby constraining its overall degrees of freedom.

Meanwhile, the pattern at the vertices also imposes limitations on the range of motion of the 9R loop:

(1) the radius of the outer center of $\triangle D_1D_4D_7$ is smaller than that of AD_i.

(2) When AB_1 and AB_8 (or other equivalent members) are collinear, they reach the limit position.

(3) In the minimum loop of a polyhedron, any two faces are coplanar.

S1.2 Three ways to increase constraints

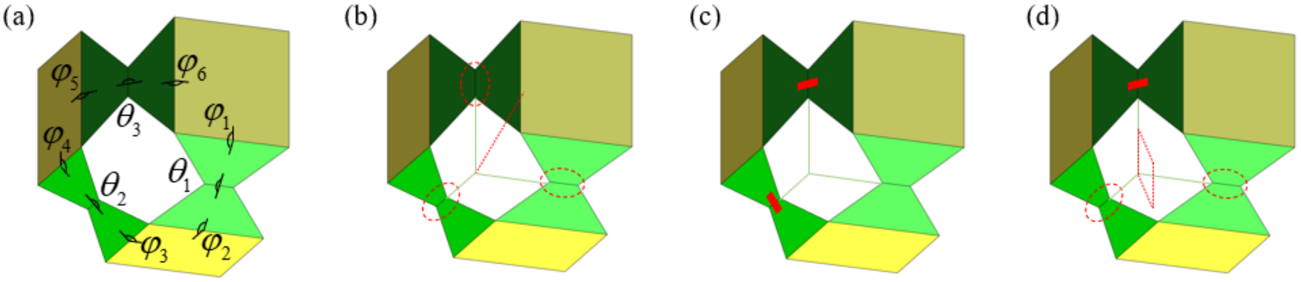

Figure S1-1. Three ways to add constraints to the 9R loop. (a) 9R loop with $\theta_1 \sim \theta_3, \varphi_1 \sim \varphi_6$. **(b)** Central symmetric constraint. **(c)** two fixed constraints. **(d)** one face symmetric constraint and one fixed constraint.

In the 9R loop, central symmetric constraint introduces the condition: $\theta_1 = \theta_2 = \theta_3$ in **Figure S1-1-**

b. Two fixed constraints applies $\theta_2 = \theta_3 = 0$ in **Figure S1-1-c** and one face symmetric constraint while one fixed constraint imposes $\theta_1 = \theta_2, \theta_3 = 0$ in **Figure S1-1-d**.

Three additional constraints are substituted into Equation S1-3. In addition, the influence of point-connection patterns on the motion range is taken into consideration. Based on these, the kinematic relationships of the three single-degree-of-freedom polyhedral are derived:

$$\text{For CP:} \quad \begin{cases} \theta_1 = \theta_2 = \theta_3 \\ \varphi_1 = \varphi_2 = \varphi_3 = \varphi_4 = \varphi_5 = \varphi_6 = 225° - \dfrac{\theta_1}{2} \end{cases} \tag{S1-4}$$

$$\theta_1 \in (100.63, 360)°$$

$$\text{For LP:} \quad \begin{cases} \theta_2 = \theta_3 = 180° \\ \varphi_1 = \varphi_2 = 225° - \dfrac{\theta_1}{2} \\ \varphi_3 = \varphi_6 = 180° - \arcsin(\dfrac{\sqrt{2}}{2}\sin\dfrac{\theta_1}{2}) \\ \varphi_4 = \varphi_5 = 90° + \arcsin(\dfrac{\sqrt{2}}{2}\sin\dfrac{\theta_1}{2}) \end{cases} \tag{S1-5}$$

$$\theta_1 \in (123.12, 360)°$$

$$\text{For FP:} \quad \begin{cases} \theta_1 = \theta_2 \\ \theta_3 = 180° \\ \varphi_1 = \varphi_4 = 360° - \dfrac{\theta_1}{2} - \arccos(-\dfrac{\sqrt{2}}{2\sin\dfrac{\theta_1}{2}}) \\ \varphi_2 = \varphi_3 = 90° - \dfrac{\theta_1}{2} + \arccos(-\dfrac{\sqrt{2}}{2\sin\dfrac{\theta_1}{2}}) \\ \varphi_5 = \varphi_6 = 135° \end{cases} \tag{S1-6}$$

$$\theta_1 \in (118.39, 262.78)°$$

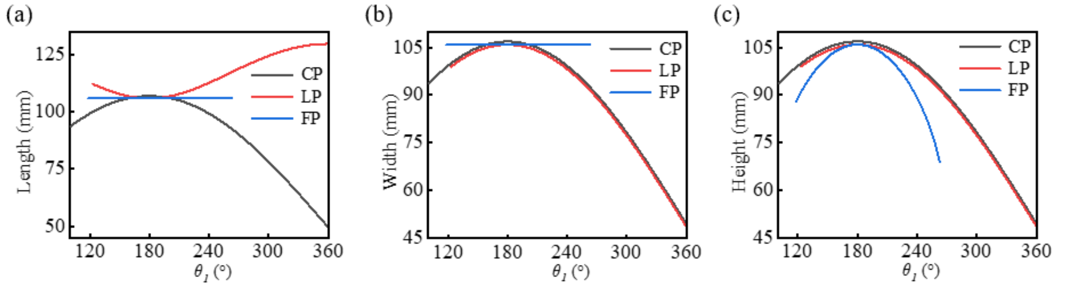

Figure S1-2. Geometric dimension changes of polyhedral origami. (a) θ_1 -length. **(b)** θ_1 -width. **(c)** θ_1 -height.

When $\theta_1 = 180°$, the three branches of motion have a common intersection point, corresponding to the bifurcation point of the polyhedron's motion.

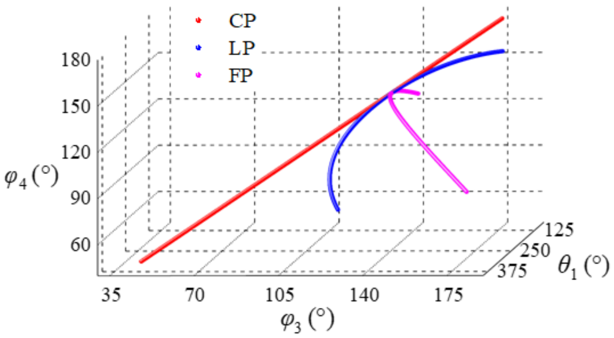

Figure S1-3. Motion bifurcation of polyhedral origami.

S2. Static analysis

Calculate the relationship between F_1 and N_1 based on the kinematic relationship of the LP path.

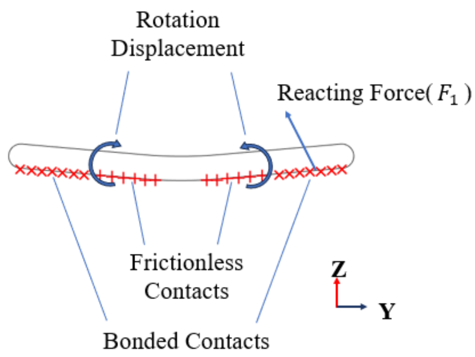

Figure S2-1. Stress Decomposition of Elastic Body

Decompose the force F_1 into its normal component F_{1N} and tangential component F_{1T} .The normal force F_{1N} is defined as positive when directed outward from the surface of the flexible body, while the tangential force F_{1T} is positive when directed away from the center of curvature. The geometric relationships lead to the following expressions:

$$\begin{aligned} F_{1N} &= -F_{1Y} \times \sin(\alpha_1 + 5°) + F_{1Z} \times \cos(\alpha_1 + 5°) \\ F_{1T} &= -F_{1Y} \times \cos(\alpha_1 + 5°) - F_{1Z} \times \sin(\alpha_1 + 5°) \end{aligned} \quad \text{(S2-1)}$$

When the polyhedron is in LP1, there are two symmetrical faces, and the bonded flexible body also satisfies these two symmetry relationships. Therefore, the internal forces of the mechanism in the X and Y directions are self balanced, and the sum of internal moments of the mechanism is 0. Only the mapping of internal forces in the Z direction can adequately represent the internal force distribution within the entire mechanism. This reflects the influence of elastic deformation in the flexible body. Decompose F_{1N} and F_{1T} into the Z direction corresponding to the coordinate system in **Figure S2-1**, and the geometric relationship can be obtained as follows:

$$F_1^Z = -F_{1T} \times \sin(\varphi_2) + F_{1N} \times \cos(\varphi_2) \tag{S2-2}$$

$$N_1 = 2F_1^Z \tag{S2-3}$$

The above analysis establishes a quantitative relationship between N_1 and F_1. From the location of flexible body adhesion, it can be inferred that $\alpha_1 = 85° - \theta_1 / 2$.The relationship between φ_2 and θ_1 as well as between φ_2 and H, are governed by the kinematic constraints illustrated Equation S1-5. These constraints form the basis for deriving the theoretical relationship between displacement and force.

S3. Poisson's ratio calculation for networking

Taking the derivative network of the LP-Rotated configuration as an example, there are k_x (or k_y) long-edge LP modules along the X (or Y) direction, $k_x, k_y \in N+$. The Poisson's ratio of the programmable LP-Rotated derived network can then be calculated as follows:

$$\begin{cases} v_{ZX} = -\dfrac{H \cdot dL}{L \cdot dH} = -\dfrac{H_2}{L_2 + k_x D_2} \cdot \dfrac{d(L_2 + k_x D_2)}{dH_2} \\ = -\dfrac{2l_1 + l_2 + l_3 + 2d_0(\sin\varphi_1 - \cos\varphi_2 - 1)}{(1+k_x)(2l_1 + l_2 + l_3) + 2d_0(\sin\varphi_1 - \cos\varphi_2 + x(\sin\varphi_4 - \cos\varphi_3) - k_x - 1)} \\ \cdot(1 + k_x \cdot \dfrac{d\sin\varphi_4 - d\cos\varphi_3}{d\sin\varphi_1 - d\cos\varphi_2}) \\ v_{ZY} = -\dfrac{H \cdot dD}{D \cdot dH} = -\dfrac{H_2}{L_2 + k_y D_2} \cdot \dfrac{d(L_2 + k_y D_2)}{dH_2} \\ = -\dfrac{2l_1 + l_2 + l_3 + 2d_0(\sin\varphi_1 - \cos\varphi_2 - 1)}{(1+k_y)(2l_1 + l_2 + l_3) + 2d_0(\sin\varphi_1 - \cos\varphi_2 + y(\sin\varphi_4 - \cos\varphi_3) - k_y - 1)} \\ \cdot(1 + k_y \cdot \dfrac{d\sin\varphi_4 - d\cos\varphi_3}{d\sin\varphi_1 - d\cos\varphi_2}) \end{cases} \tag{S3-1}$$

By substituting the kinematic relationship of the LP module into Equation S1-5, the Poisson's ratio of the derived network can be obtained. Since the values of k_x and k_y are within the range of positive integers, the Poisson's ratio of the derived network is a specific set.

For different combinations of derivative networks, set a total of n derivative networks in the X and Y directions, and the i-th derivative network has $k_x = k_{x_i}, k_y = k_{y_i}$. Calculate the Poisson's ratio of the final combination:

$$
\begin{cases}
v_{ZX} = -\dfrac{H \cdot dL}{L \cdot dH} = -\dfrac{H_2}{nL_2 + \sum_{i=1}^{n} k_{x_i} D_2} \cdot \dfrac{d(nL_2 + \sum_{i=1}^{n} k_{x_i} D_2)}{dH_2} = -\dfrac{H_2}{dH_2} \cdot \dfrac{d(L_2 + \overline{k_x} \cdot D_2)}{L_2 + \overline{k_x} \cdot D_2} \\
= -\dfrac{2l_1 + l_2 + l_3 + 2d_0(\sin\varphi_1 - \cos\varphi_2 - 1)}{(1+\overline{k_x})(2l_1 + l_2 + l_3) + 2d_0(\sin\varphi_1 - \cos\varphi_2 + \overline{k_x}(\sin\varphi_4 - \cos\varphi_3) - \overline{k_x} - 1)} \\
\cdot(1 + \overline{k_x} \cdot \dfrac{d\sin\varphi_4 - d\cos\varphi_3}{d\sin\varphi_1 - d\cos\varphi_2}) \\
v_{ZY} = -\dfrac{H \cdot dD}{D \cdot dH} = -\dfrac{H_2}{nL_2 + \sum_{i=1}^{n} k_{y_i} D_2} \cdot \dfrac{d(nL_2 + \sum_{i=1}^{n} k_{y_i} D_2)}{dH_2} = -\dfrac{H_2}{dH_2} \cdot \dfrac{d(L_2 + \overline{k_y} \cdot D_2)}{L_2 + \overline{k_y} \cdot D_2} \\
= -\dfrac{2l_1 + l_2 + l_3 + 2d_0(\sin\varphi_1 - \cos\varphi_2 - 1)}{(1+\overline{k_y})(2l_1 + l_2 + l_3) + 2d_0(\sin\varphi_1 - \cos\varphi_2 + \overline{k_y}(\sin\varphi_4 - \cos\varphi_3) - \overline{k_y} - 1)} \\
\cdot(1 + \overline{k_y} \cdot \dfrac{d\sin\varphi_4 - d\cos\varphi_3}{d\sin\varphi_1 - d\cos\varphi_2})
\end{cases}
\tag{S3-2}
$$

Among them, $\overline{k_x} = \dfrac{\sum_{i=1}^{n} k_{x_i}}{n}, \overline{k_y} = \dfrac{\sum_{i=1}^{n} k_{y_i}}{n}$, according to the range of values for k_x and k_y, $\overline{k_x}, \overline{k_y} \geq 1$.

Similar to the LP-Rotated network, the other types of networks in **Figure 3b** can also be derived through specific transformations and subsequently recombined. **Figure S3-1** shows these derived networks, and **Figures S3-2** to **S3-5** demonstrate four possible methods for their recombination.

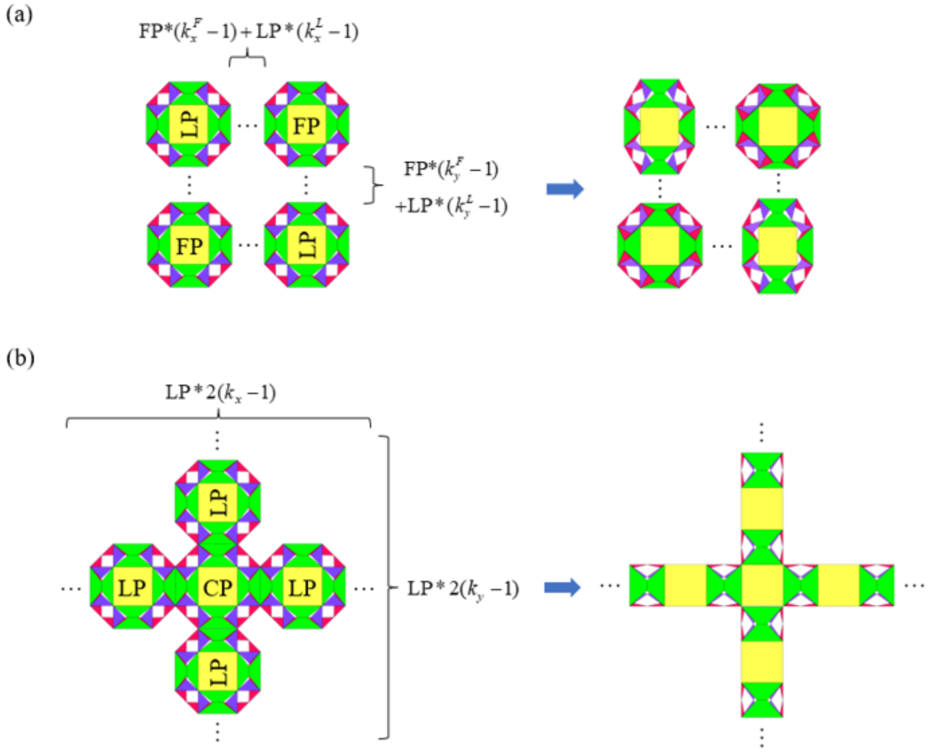

Figure S3-1. Two ways to make derived networking. (a) Adding LP, FP module to LP, FP-Mixed-2. **(b)** Adding LP module to CP, LP-Mixed network.

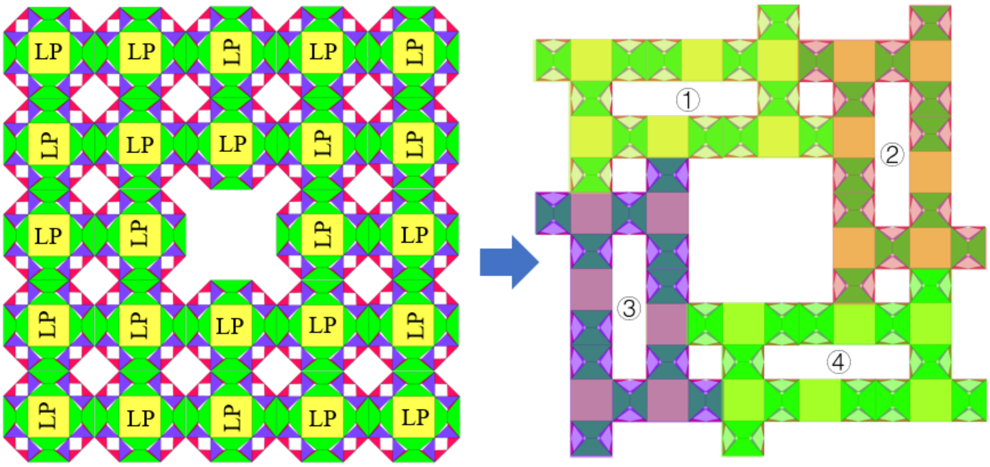

$\overline{k_x} = \overline{k_y} = 1.5$

Figure S3-2. LP-Rotated Networking Systems.

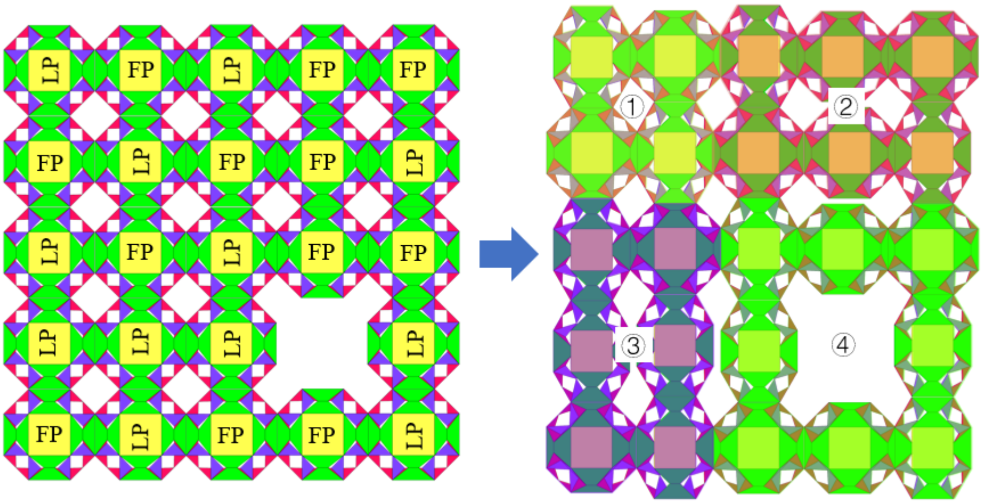

$\overline{k_x^L} = \overline{k_y^L} = 1, \overline{k_x^F} = \overline{k_y^F} = 1.5$

Figure S3-3. LP, FP-Mixed-1 Networking Systems.

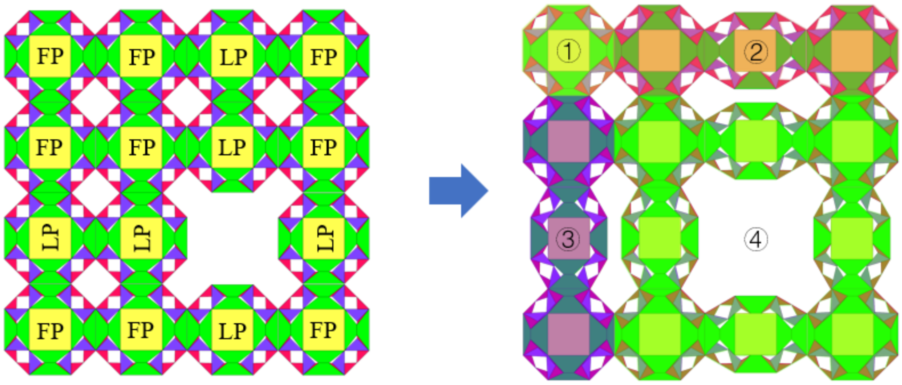

$\overline{k_x^L} = \overline{k_x^F} = \overline{k_y^L} = \overline{k_y^F} = 2$

Figure S3-4. LP, FP-Mixed-2 Networking Systems.

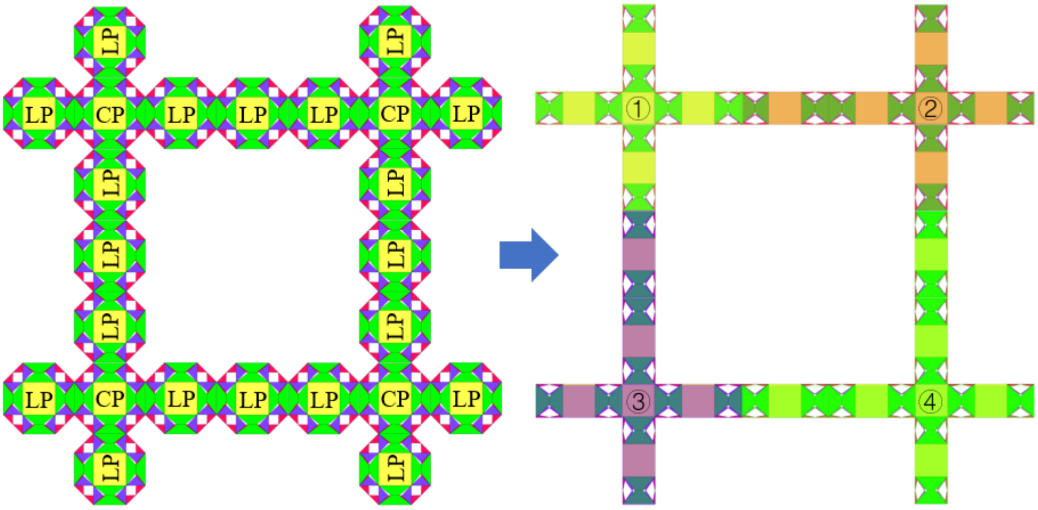

$\overline{k_x} = \overline{k_y} = 1.25$

Figure S3-5. CP, LP-Mixed Networking Systems.

Figures S3-6 to **S3-8** show the Poisson's ratios that can be achieved by combining these derived networks:

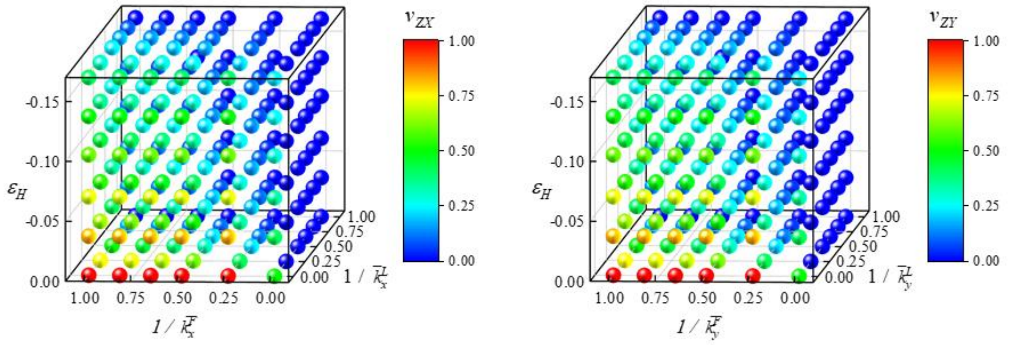

Figure S3-6. v_{ZX}, v_{ZY} **of LP, FP-Mixed-1 derived network recombination.**

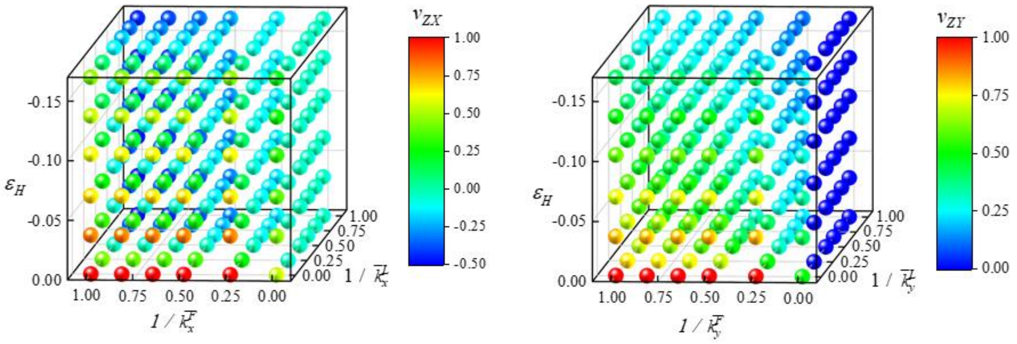

Figure S3-7. v_{ZX}, v_{ZY} **of LP, FP-Mixed-2 derived network recombination.**

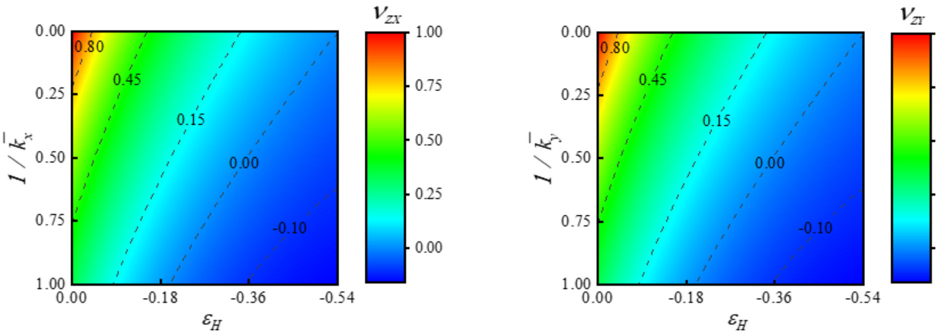

Figure S3-8. ν_{ZX}, ν_{ZY} **of CP, LP-Mixed derived network recombination.**

S4. Fold design of polyhedral origami

The crease is constructed by placing a 0.1 mm thick high-strength polyimide double-sided tape between two acrylic sheets. The design considers the distinct roles of inner and outer creases in the folding process. To implement this, one of the acrylic layers (either upper or lower) is fully cut, while the other undergoes selective material removal. This configuration forms a rotational joint within the folding mechanism and enables unidirectional folding in **Figure S4-1**.

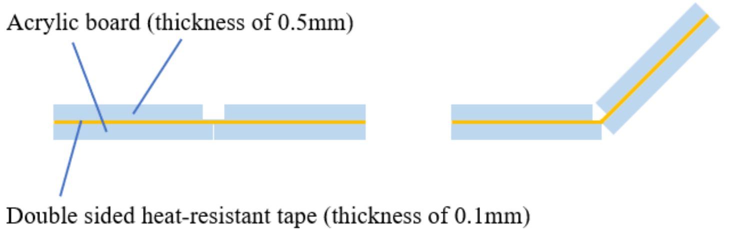

Figure S4-1. Design of creases and rigid surfaces.

The cutting patterns corresponding to the lower and upper acrylic layers are presented in **Figure S4-2a** and **Figure S4-2b** respectively. The removal of the blue line material is to leave space for the subsequent connection of the flexible body. The cutting pattern of the double-layer acrylic sheet after pasting is showed in **Figure S4-2-c**. After the adhesive layer is applied, the polygonal folding mechanism is cut from the acrylic sheet and the excess material is subsequently removed. The small circular holes are used to leave through holes for the fastening of the flexible body.

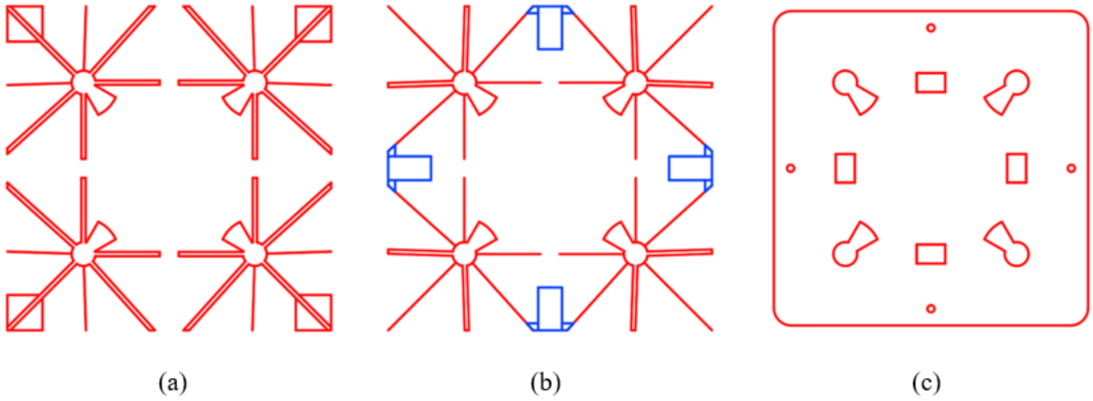

Figure S4-2. Cutting pattern of acrylic board. (a) Lower layer acrylic board cutting pattern. **(b)** Upper layer acrylic board cutting pattern. **(c)** Cut pattern after pasting.
